# Genomic analysis association of tolerance to heat stress in subtropical Egyptian goats Raised in hot dry environment

**DOI:** 10.1186/s12864-025-11748-x

**Published:** 2025-06-11

**Authors:** Adel M. Aboul-Naga, Alsamman M. Alsamman, Sherif Melak, Ahmed E. Nassar, Taha H. Abdelsabour, Layaly Gamal, Monica Adley, Mohmed H. Elshafie

**Affiliations:** 1 Animal Production Research Institute, Agriculture Research Center (ARC), P.O. 12619, Cairo, Egypt; 2 Agricultural Genetic Engineering Research Institute (AGERI), ARC, Cairo, Egypt; 3 International Center for Agricultural Research in the Dry Areas (ICARDA), P.O. 12619, Giza, Egypt

**Keywords:** Heat stress tolerance, Subtropical egyptian goat, Genomic analysis, GWAS

## Abstract

The study investigated subtropical Egyptian goat populations (382 does) from different hot dry ecological zones (Upper Egypt, Coastal Zone of Western Desert, and New Valley Desert Oasis) to identify genes associated with tolerance to heat stress. Animals were encouraged to walk for 7 km under direct solar radiation from 12:00 to 14:00 pm in July and August (imitating summer grazing on poor pasture under hot dry conditions). Temperature Humidity Index (THI) ranged from 98.6 to 109.3, indicating that the animals were under severe heat stress. Physiological parameters were measured at rest (7:00 am) and after exposure to heat stress (14.00 pm). Animal heat tolerant index (AHTI) was estimated from their response in the four main physiological parameters ranged from (0: high tolerant to 4: low tolerant). The GWAS analysis revealed 90 marker SNPs associated with heat stress in 108 genotypes of Egyptian goat. Ninety markers are found in forty-seven distinct genes distributed across the genome. In terms of the markers (SNPs) that have direct effect on these traits via homozygous alleles, twenty-eight SNPs are connected to heat stress. The snpeff approach revealed that *KDM6A*,* TRPM3*,* USP54*,* GLTSCR2*,* NAALADL2*,* GATAD2A*,* CTNNA2*,* LOC102175876*,* ZBTB8A*,* ETNPPL*,* LRRC43*,* SNTB1*,* RPS6KA5*, and *ARHGAP26* genes influence tolerance to heat stress. These genes offer crucial insights into the biological mechanisms that enhance resilience to elevated temperature conditions. The studied subtropical Egyptian goat breeds showed a high ability to tolerate heat stress and identifying these genomic loci can be utilized to monitor and control tolerance of subtropical goat to heat stress, while maintaining their production performance.

**Supplementary Information:**

The online version contains supplementary material available at 10.1186/s12864-025-11748-x.

## Introduction

Climate changes caused by the global warming is considered a major threat to animals’ production in many parts of the world [1]. It has negative impact on both feed availability and livestock production; reducing milk yield and quality, as well as meat production [2]. It causes behavioral and physiological changes of the animals, including decreasing feed intake and metabolic activity, and consequently their production [3]. Heat stress (HS) directly suppresses immune function in the animals, making them more susceptible to diseases [4]. Climate changes has indirect effects on the availability of feed and fodder resources (grasses, forages, and grains), as well as the severity and spread of diseases [5]. Livestock responds to changing environments by varying their phenotypic and physiological parameters. To maintain livestock production in a climate change affected environment, the animals must be genetically compatible and capable of surviving stressful conditions [2].

One of the main reasons beyond the wide spread of goats globally, is their resilience to changing environments, and the wide feeding regimes they are maintained. There are about 1.15 billion goats on the planet, around 95% of them in Asia, and Africa [6]. Goats can resist HS and water scarcity for extended periods of time. They are adaptable to harsh climatic and geophysical situations in which cattle and sheep cannot thrive [7]. They efficiently consume poor-quality forages and walk long distances in search of food. Their distinctive feeding habits make it easier to select diets that fulfill their needs [8]. The widely distributed goat in Egypt is the Baladi goats (means local in Arabic) found primarily in Delta, the second widely distributed breed is Saidi goats along Upper Egypt. Barki is a desert goat, extended all over the coastal zone of Western Desert, it has a straight physique, a small head, medium and drooping ears, long black hair with white patches. These local breeds are of great economic and cultural importance in the rural areas, they are raised for both milk and meat. Local goats are good fertile raised primarily for milk and kid production, they produce moderate amounts of milk, which often used for homemade cheese and other dairy products. Saidi goat is well-adapted to hot, dry weather and shifting climates, they are good prolific goats and daily milk yield ranges from 300 to 1150 g. Wahati goats are distinguished by their heat endurance, small body size, black glossy hair, and milk yields ranging from 30 to 40 kg throughout the lactation period [9].

Goat genome is 2.66 Gb in size, with an N50 of 3.06 Mb [10]. It has 30 chromosomes and 43.15% GC content [11], contains 22,175 protein-coding genes. Since their specific morphological and physiological performance, goats have a wide spectrum of ecological adaptation [12]. Number of genes involved in goats tolerance to HS had been identified, including growth hormone receptor (*GHR*), insulin-like growth factor-1 *(IGF-1*), leptin (*LEP*), and thyroid hormone receptor (*THR*), which are linked to HS influence on their physiological growth pathways [13]. Approximately fifty different genes change their expression during thermal stress, includes: *IL-6-Interleukin 6*, *Mitogen Activated Protein Kinase 14*, *NOS 2-Nitric Oxide Synthase 2*, *NOS 3-Nitric Oxide Synthase 3*, *UCP 3-Uncoupling Protein 3*, *CRP-C Reactive Protein* and *ATP1A1-Na+/K + transporting subunit alpha A1* [14]. *Hsp70* expression levels were significantly high in the tropical goats during summer, which plays an important role in their tolerance to thermal stress under hot climate [15]. The expression of *GST*,* CAT*,* HSP70*,* BLF* and *LACT* genes showed higher level of expression in high tolerant Egyptian goats and lower level in low tolerant individuals [16]. Studying the genetic diversity of 17 million single nucleotide polymorphism markers (SNPs) in Du’an goat, revealed selective signals associated with adaptive traits including immunological resistance (*serpin cluster*, *INFGR1*, *TLR2*, and *immune-related pathways*), body size (*HMGA2*, *LCOR*,* ESR1*, and *cancer-related pathways*), and heat tolerance (*MTOR*, *ABCG2*, *PDE10A*, and *purine metabolism path way*) [17]. The stimulation of transcription of “heat shock proteins” (HSPs) in response to thermal stress is well recognized [18]. The expression of HSPs may serve as a predictor of animal’s ability to adapt extreme climatic stress [19].

SNP genotyping is an appropriate tool for investigating goats’ demographic history and estimating their genetic diversity, as well as their phylogenetic relationship with other breeds [20]. Findings about livestock biodiversity provides information about its demographic distribution in the world, their indigenous home and adaptability to the prevailed conditions [21]. Goat biodiversity conservation is exceedingly crucial since there are so many different breeds. However, comparing to other domesticated animals, goats have the least well-documented biodiversity [22].

Egyptian goats, recognized for their resilience in harsh climatic conditions, represent a valuable genetic resource for comprehending heat stress tolerance, meanwhile the genetic mechanisms underlying this feature remain poorly understood. Prior studies predominantly focused on behavioral and physiological responses to HS, with limited exploration of the genetic base that confer this resilience. The current study aims to identify the genetic markers and physiological characteristics associated with HS tolerance in Egyptian goat breeds under various hot dry environments, in order to enhance our understanding of the genetic mechanisms involved in their tolerance to HS.

## Materials and methods

### The studied agro-ecological zones

**Fig. 1:**
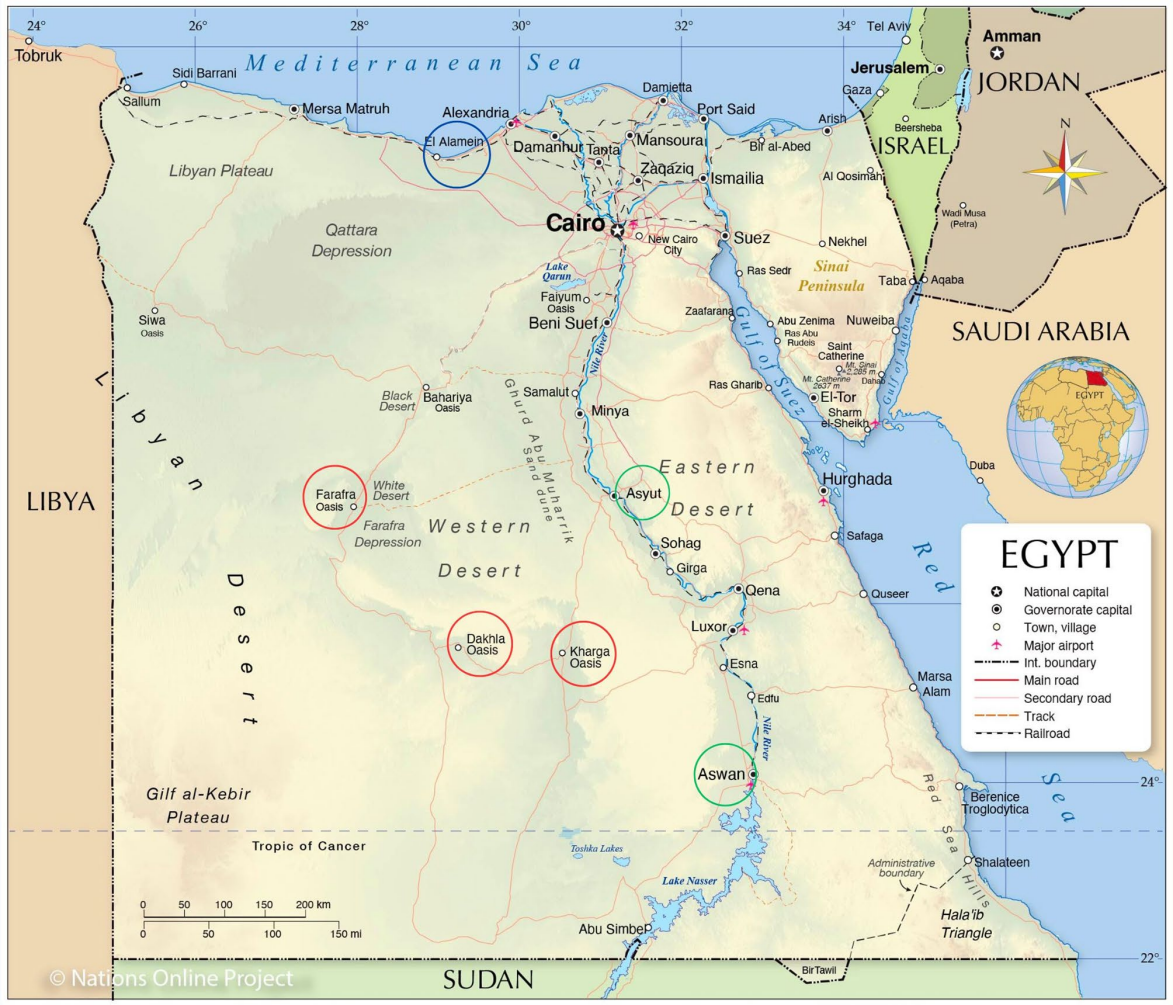
Location of the studied Egyptian goat breeds; Coastal Zone of Western Desert (blue), New Valley desert oasis (red), and Upper Egypt (green)

National program was initiated in 2009 by Animal Production Research Institute (APRI), Agriculture Research Center (ARC), to assess tolerance capacity of local Egyptian goat populations raised under harsh environments to HS. Three hots dry agro-ecological zones were involved in the study (Fig. 1).

- The first region is the Coastal Zone of the Western Desert (CZWD), extended from Alexandria (Egypt) east to Libyan board west, with annual rainfall of less than 140 mm, having 3-months of poor-quality ranges in the winter, and sparse vegetation in the long summer.
- The second region is the desert oases in the New Valley (NV) governorate, southwest of Egypt, extended from the Nile Valley in the east to the Libyan borders in the west, and from Matrouh governorate in the north to Sudan border in the south. It contains three major desert oases: Dakhla, Kharga and Farafra. The climatic conditions are hot temper summer (ambient temperature under shed reach 50^°^C), scarce rainfall (2–10 mm/ annually) and highly intensive solar radiation, ambient temperature varied widely between day and night, often exceeds 20^°^C.
- The third region is Upper Egypt, hot dry area extended from Giza governorate north to Sudan border in the south (latitudes: 22^°^ south to 29^°^ north). It is characterized by very hot summers, cold winter nights, and scarce rainfall (15 mm/annually). Ambient temperature varied widely between day and night. The prevailing agro-ecological system is intensive agriculture of more than crop/year, and mixed crop-livestock production system. Longitudinal climatic data of the three different regions under study are shown in table S1.

### Breeds and populations

Three hundred eighty-two adult females from three local populations, raised in the three studied regions under hot dry harsh conditions, were investigated. The first population was 232 Barki goats from the CZWD. They are hardly animals, well suited to the arid conditions and scarce vegetation. The second population composed of 100 Wahati goats, from three desert oases, extended over 500 km apart. Their predominant color is black, though various lighter tints may be observed, long silky hair and horny Males [23]. The third population consists of 50 Saidi goats, indigenous to Upper Egypt, they are raised in small herds under intensive agriculture system. They are known for their adaptation to the severe hot and dry environment of Upper Egypt.

### Experimental procedure

Animals involved in the heat tolerance investigation were non-pregnant and non-lactating does, aged two to six years. Varied breeders in each region own the animals, and written informed consent was obtained from the owners for the participation of their animals in the study. They generally fed Egyptian clover in the winter and concentrates plus hay in the summer. They had free access to water except during exposure to heat stress. To assess the physiological responses to HS, each animal served as its own control. Pre-HS measurements were taken at rest (7:00 am) and post-HS measurements were taken after exposure to HS (14:00 pm). Animals were encouraged to walk for seven kilometers under direct solar radiation from 12:00 to 14:00 pm in July and August (imitating summer grazing on poor pasture, under direct solar radiation). The within-subject design allows for the comparison of physiological parameters before and after exposing to HS, thereby controlling individual variability and providing more accurate assessment of each animal’s response. Metrological parameters: dry-bulb temperature (DBT), relative humidity (RH)) were taken at rest (7:00 am) and after exercise at 14:00 pm. Temperature-humidity index (THI) was estimated according to the equation of [24]. THI varied from 98.6 to 109.3, indicating that the animals were suffering from severe heat stress (Table 1).

$$\eqalign{ THI = & \left({\left({DBT1.8} \right) + 32} \right) - \left({\left({0.55 \times \left({RH/100} \right)} \right)} \right) \cr & \times \left({\left({DBT \times 1.8} \right) + 32} \right)-58 \cr} $$

Physiological parameters were measured at rest (7:00 am) and after exposure to heat stress (14:00 pm). Rectal temperature (RT) and skin temperature (ST) were measured using clinical (°C) and infrared thermometer, respectively. Respiration rate (RR, res/min) and gas volume (GV, L/min) were measured using Harvard dry gas meter. Values of RT, ST, RR, and GV were used to develop animal heat tolerant index (AHTI) according to [16]. Animals were scored 1 if changes in any parameter were greater than 2 standard deviations of their resting value; otherwise, they scored zero. AHTI ranged from zero (high tolerant) to four (low-tolerant) animals.

**Table 1:** Climatic parameters at 7:00 am and 14:00 Pm at different regions

Agro-ecological region	THI (7:00 am)	THI (14:00 pm)
Coastal Zone, Western Desert (CZWD)	71.9	105.8
Upper Egypt (UE)	75.8	107.2
New valley Desert Oasis (NV)	81.1	110.0

### DNA sampling and genomic analysis

Blood samples were collected from 108 mature goats and DNA was extracted using the QI Amp MINI kit procedure, directly after exposure to heat stress. The samples are grouped into three populations: 50 Saidi goats (15 from Assuit, 23 from Aswan, and 12 from Malawi); 27 Barki (from Matrouh governorate); and 31 Wahati goats from the New Valley governorate (9 from Dakhla, 14 from Farafra and 8 from Kharga oases), based on the breed and geographic location. DNA sampling is related to the population size of the breed in the country. Genomic analysis was performed at the LABOGENA DNA laboratory within the “PERFORM” project using the genotypes on microchips (50 kb) for Caprine by Gene Seek. SNPs with call rates < 90%, values of chi-square tests for HWE less than10^-6^ were filtered and removed with bioinformatics techniques. The analysis yielded 52,280 SNP markers after filtering.

### Genomic association and statistical analysis

Using the vcf2gwas tool, genomic analysis was carried out for tolerance to HS, vcf2gwas is a Python API for identifying GEMMA according to [25]. Genome Efficient Mixed Model Association (GEMMA) uses univariate linear mixed model and Bayesian sparse linear mixed model were used to assess marker relationships with tolerance to HS [26, 27]. In the Manhattan plot, SNPs with *p*-value of 0.001 were identified as associated with HS. The Q-Q plot analysis in the R environment was used to evaluate the observed data’s goodness-of-fit to theoretical distribution in which each observation is represented by a symbol [28]. The boxplot function in R- package (v4.0.3) was used to display SNPs effects on tolerance to HS via homozygous alleles for SNP-annotation. Using a total of 90 annotated SNPs, snpeff was applied to analyze the genetic variation influence on genes and proteins throughout the festa and annotated (GFF) Capra circus (GCF 001704415.1 ARS1) genome [29]. R environment (v4.0.3) mapped SNPs locations on compacted genes based on snpeff output for additional categorization and description of SNPs influences on genes and the enrichment analysis of the identified genes was performed by Shiny GO website (http://bioinformatics.sdstate.edu/go/) (Table S2). Genetic variations within breeds and locations were assessed based on the significant 90 SNPs associated with AHTI using principal component analysis (PCA) by Origin (Origin Ro, Version 2024. Origin Lab Corporation, Northampton, MA, USA.) to show the genetic similarity between breeds according to tolerance to HS. Linear regression model with pairwise comparison method was used to identify SNP-SNP combinations by SAS [30]. The datasets generated and analyzed from the study can be found in the following online repository (https://zenodo.org/records/14885406).

## Results

### Physiological response of goats to heat stress

Changes in the physiological parameters of the studied goat populations exposed to HS were statistically highly significant (*P* ≥ 0.01) (Table 2). Respiration rate (RR) showed the largest significant changes among the studied physiological parameters, with values exceeding 400.7% of their values at rest in Barki goats, 147.2% of their values at rest for Wahati goats and 208.7% of their values at rest for Saidi goats. Changes in gas volume were statistically highly significant (*P* ≥ 0.01), it was the largest in Barki goats 225%, followed by Saidi goats 123.1%, and the least in Wahati goats (85.7%). The changes in rectal and skin temperature (RT and ST) with HS were less recognized than that of RR and GV, ranging from 2.8 to 4.3% for RT and from 7.9 to 24.5% for ST. The largest breed variation was found in the RT and GV parameters (Table 2). Breed, and animal variation (within breed) were the main contributing factors to the changes in the physiological parameters of the animals with HS (Table 3). Individual variation was highly significant (*P* ≥ 0.01) in RR, and significant in GV (*P* ≥ 0.05). Changes in the RR of the subtropical goats when exposed to HS seems to be the most reliable criteria to differentiate animals tolerant to HS from non-tolerant animals. Saidi goats showed significantly lower heat tolerance index than both Barki and Wahati populations (2.4 vs. 3.0 and 3.3, respectively), indicating that the Saidi goats are more adapted to HS under hot dry conditions, than both Barki and Wahati goats. It is interesting to note that Wahati goats of the desert oases showed high percentage of heat-tolerant animals than Saidi (Upper Egypt) and desert Barki goats (14 vs. 12 and 8.6%, respectively), which indicates good room for selection for heat tolerant animals in the Wahati goats (Tables 3 and 4).

**Table 2:** Changes in physiological parameters of subtropical Egyptian goats exposed to HS

Items	Parameters^*^	Barki goats (232 does)	Saidi goats (50 does)	Wahati goats (100 does)
Physiological Parameter at 7:00 am	RT (^*°*^C)	39.1 *±* 0.03	38.9 *±* 0.08	38.9 *±* 0.05
	ST (^*°*^C)	36.9^a^ *±* 0.13	33.3^c^ *±* 0.27	35.5^b^ *±* 0.16
	RR (res./min)	26.7^a^ *±* 0.47	20.6^c^ *±* 1.20	23.1^b^ *±* 0.71
	GV (L/min)	2.8^a^ *±* 0.08	1.3^c^ *±* 0.19	2.1^b^ *±* 0.11
Physiological Parameter at 14:00 pm	RT (^*°*^C)	1.7^a^ *±* 0.04	1.2^b^ *±* 0.11	1.1^b^ *±* 0.06
	ST (^*°*^C)	2.9^b^ *±* 0.18	3.2^b^ *±* 0.38	8.7^a^ *±* 0.23
	RR (res./min)	107^a^ *±* 2.68	43^b^ *±* 6.89	34^b^ *±* 4.08
	GV (L/min)	6.3^a^ *±* 0.32	1.6^b^ *±* 0.83	1.8^b^ *±* 0.49
	AHTI	3.0^b^ *±* 0.02	2.4^c^ *±* 0.16	3.3^a^ *±* 0.09
Changes (%)	RT (^*°*^C)	4.3	3.1	2.8
	ST (^*°*^C)	7.9	9.6	24.5
	RR	400.7	208.7	147.2
	GV	225.0	123.1	85.7

^*^RT: rectal temperature, ST: skin temperature, RR: respiration rate, GV: gas volume, and AHTI: animal heat tolerance index

**Table 3:** ANOVA for changes in physiological parameters of Egyptian goats exposed to HS

SOV^*^	DF	SS				
		RT	ST	RR	GV	AHTI
Breed	2	12.0^**^	986^**^	189,199^**^	925^**^	9.6^**^
ID (breed)	273	0.44	6.4^**^	1675	26.4^*^	0.69
Residual	91	0.38	1.8	1638	17.9	0.89

^*^ SOV: source of variance, DF: degree of freedoms, RT: rectal temperature, ST: skin temperature, RR: respiration rate, GV: gas volume, and AHTI: animal heat tolerance index, * *P* < 0.05 and ** *P* < 0.01

**Table 4:** Distribution of Barki, Saidi and Wahati goats according to their heat tolerance (%)

Heat stress	Barki	Saidi	Wahati
High	8.6	12.0	14.0
Medium	65.9	71.4	45.0
Low	25.4	17.1	41.0

### Genomic association analysis

**Fig. 2:**
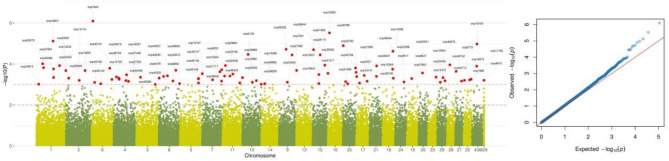
Manhattan plot of the genome analysis showing SNPs associated with tolerance to heat stress (HS). Red points are SNPs have a significant association with HS. The QQ plots are used to compare the quantile distribution of observed p-values (on the y-axis) to the quantile distribution of expected p-values of SNPs

The genomic association analysis involved 108 genotypes and 52,280 marker-SNPs. The results revealed 90 marker-SNPs associated with tolerance to HS distributed across the genome (Fig. 2). Chromosome 1 had the highest number of SNPs associated with tolerance to HS, with eight identified SNPs, while Chromosomes 12, 19, 23, 24, 26 and 28 have only one identified SNP per chromosome (Table 5). The strongest association with HS was observed for the SNPs located on Chromosomes 6, 16, and 1, at positions 7,672,687, 8,638,832, and 88,790,472 with *p*-values of $\:8.04*{10}^{-7}$, $\:{3.02*10}^{-6}$, and $\:{7.62*10}^{-6}$, respectively. The SNP effect for tolerance to heat stress ranges from 0.057 to 0.5 of the measured unit, with SNP19073, SNP38828 having the lowest values and snp43427, snp48343, snp38199, and snp40201 having the highest values. The most frequent mutation types were the AG (45 SNPs), GA (39 SNPs), CA (4 SNPs), and AC (2 SNPs). Among those, Chromosome 1 exhibited the largest number of identified SNPs (8 SNPs), followed by Chr8 and Chr4 with 6 SNPs and Chr6, 7, 11, and 15 each with 5 SNP.

**Table 5:** SNPs association with heat stress tolerance in Egyptian goat, chromosome (Chr.), position (PS), *p*-value scores (*p* value), alleles (Al.) and effect of SNPs on trait by unit

SNP	Chr.	PS	Al.	*p* value	Effect	SNP	Chr.	PS	Al.	*p* value	Effect
snp7643	Chr6	7,672,687	A > G	8.04 × 10^− 07^	0.207	**snp9637**	Chr23	42,943,557	G > A	4.36 × 10^− 04^	0.136
snp16282	Chr16	8,638,832	A > G	3.02 × 10^− 06^	0.421	**snp22586**	Chr1	91,603,605	A > G	4.51 × 10^− 04^	0.114
snp44801	Chr1	88,790,472	G > A	7.62 × 10^− 06^	0.129	**snp40201**	Chr3	23,271,713	A > G	4.43 × 10^− 04^	0.486
snp19103	SCAF1	28,757,795	G > A	1.05 × 10^− 05^	0.371	**snp18981**	Chr10	619,084	A > G	4.60 × 10^− 04^	0.407
snp48788	Chr20	2,904,444	G > A	1.27 × 10^− 05^	0.129	**snp22430**	Chr29	30,896,627	A > G	4.63 × 10^− 04^	0.221
snp55332	Chr9	38,107,900	G > A	1.90 × 10^− 05^	0.257	**snp6478**	Chr8	2,768,166	A > G	4.83 × 10^− 04^	0.293
snp36644	Chr15	5,720,149	A > G	2.00 × 10^− 05^	0.214	**snp23098**	Chr18	57,625,693	A > G	4.94 × 10^− 04^	0.1
snp15096	Chr18	55,684,910	A > G	2.38 × 10^− 05^	0.371	**snp14115**	Chr2	14,684,639	G > A	4.91 × 10^− 04^	0.443
snp5109	Chr10	30,979,908	A > G	3.37 × 10^− 05^	0.186	**snp56754**	Chr4	28,684,280	G > A	4.87 × 10^− 04^	0.307
snp28114	Chr15	40,980,610	A > G	3.57 × 10^− 05^	0.45	**snp40147**	Chr5	99,882,273	A > G	5.02 × 10^− 04^	0.371
snp7931	Chr9	69,923,744	G > A	3.60 × 10^− 05^	0.393	**snp20241**	Chr19	26,420,506	A > G	5.11 × 10^− 04^	0.314
snp29273	Chr1	33,186,052	A > G	9.67 × 10^− 05^	0.221	**snp40145**	Chr5	99,819,492	A > G	5.27 × 10^− 04^	0.357
snp36076	Chr28	16,081,886	G > A	1.10 × 10^− 04^	0.086	**snp11765**	Chr25	40,337,682	A > G	5.48 × 10^− 04^	0.286
snp51992	Chr15	65,810,907	G > A	1.14 × 10^− 04^	0.129	**snp16357**	Chr4	30,071,577	A > G	5.54 × 10^− 04^	0.279
snp29891	Chr11	16,075,021	G > A	1.19 × 10^− 04^	0.121	**snp40151**	Chr6	108,829,907	G > A	5.68 × 10^− 04^	0.45
snp13332	Chr1	123,373,118	G > A	1.20 × 10^− 04^	0.3	**snp7980**	Chr25	32,413,233	A > G	5.92 × 10^− 04^	0.1
snp38421	Chr21	38,115,540	G > A	1.24 × 10^− 04^	0.193	**snp59658**	Chr7	56,262,899	A > G	5.86 × 10^− 04^	0.307
snp19073	SCAF2	27,104,601	A > G	1.27 × 10^− 04^	0.057	**snp8772**	Chr25	4,673,607	A > G	6.21 × 10^− 04^	0.35
snp47064	Chr1	38,098,862	G > A	1.51 × 10^− 04^	0.393	**snp4910**	-	894	A > G	6.22 × 10^− 04^	0.364
snp50782	Chr20	68,438,400	G > A	1.57 × 10^− 04^	0.443	**snp25209**	Chr2	71,132,300	G > A	6.28 × 10^− 04^	0.15
snp59519	Chr6	97,320,058	G > A	1.61 × 10^− 04^	0.279	**snp14098**	Chr10	74,860,568	A > G	6.46 × 10^− 04^	0.321
snp22581	Chr1	91,381,905	G > A	1.74 × 10^− 04^	0.407	**snp27352**	Chr4	65,594,529	G > A	6.40 × 10^− 04^	0.314
snp38424	Chr18	54,432,875	A > G	1.79 × 10^− 04^	0.286	**snp12564**	Chr29	41,642,591	G > A	6.56 × 10^− 04^	0.464
snp9053	Chr7	104,895,355	G > A	1.90 × 10^− 04^	0.386	**snp40715**	Chr27	33,547,955	G > A	6.80 × 10^− 04^	0.179
snp27482	Chr12	2,590,013	A > G	2.05 × 10^− 04^	0.357	**snp21717**	Chr7	103,992,457	C > A	6.81 × 10^− 04^	0.286
snp30910	Chr8	91,793,520	A > G	2.02 × 10^− 04^	0.421	**snp6853**	Chr8	76,118,406	G > A	6.68 × 10^− 04^	0.1
snp55420	Chr1	156,977,663	C > A	2.09 × 10^− 04^	0.243	**snp12364**	Chr17	58,187,128	A > C	6.91 × 10^− 04^	0.329
snp16805	Chr2	102,167,399	G > A	2.07 × 10^− 04^	0.186	**snp54048**	Chr4	79,989,673	A > G	7.01 × 10^− 04^	0.114
snp48152	Chr27	4,930,875	G > A	2.27 × 10^− 04^	0.286	**snp39502**	Chr20	15,390,166	A > G	7.20 × 10^− 04^	0.129
snp15564	Chr7	95,048,654	C > A	2.38 × 10^− 04^	0.279	**snp9511**	Chr24	47,390,902	G > A	7.50 × 10^− 04^	0.371
snp45768	Chr20	71,397,347	A > G	2.54 × 10^− 04^	0.207	**snp53002**	Chr10	53,403,332	G > A	7.77 × 10^− 04^	0.207
snp27851	Chr26	21,529,500	A > G	2.75 × 10^− 04^	0.157	**snp15472**	Chr29	16,442,833	A > G	7.73 × 10^− 04^	0.1
snp41517	Chr16	746,305	A > G	2.85 × 10^− 04^	0.357	**snp24932**	Chr9	44,815,969	A > G	7.72 × 10^− 04^	0.221
snp10107	Chr7	827,494	A > G	2.96 × 10^− 04^	0.329	**snp49160**	Chr6	16,880,306	A > G	7.87 × 10^− 04^	0.464
snp48343	Chr11	55,737,577	G > A	3.08 × 10^− 04^	0.5	**snp22868**	Chr11	92,816,509	A > G	8.32 × 10^− 04^	0.457
snp16931	Chr15	34,072,378	A > G	3.25 × 10^− 04^	0.45	**snp40146**	Chr5	99,849,530	A > G	8.39 × 10^− 04^	0.3
snp27449	Chr4	69,619,376	G > A	3.36 × 10^− 04^	0.264	**snp2427**	Chr17	18,032,383	A > C	8.51 × 10^− 04^	0.35
snp16141	Chr17	43,053,452	G > A	3.62 × 10^− 04^	0.207	**snp16942**	Chr15	34,040,018	A > G	8.93 × 10^− 04^	0.436
snp43427	Chr8	46,845,710	G > A	3.74 × 10^− 04^	0.5	**snp38828**	Chr14	19,031,193	G > A	9.11 × 10^− 04^	0.057
snp23746	Chr11	11,252,041	A > G	3.85 × 10^− 04^	0.257	**snp20152**	Chr6	49,470,441	A > G	9.17 × 10^− 04^	0.257
snp20506	Chr11	40,109,350	G > A	3.87 × 10^− 04^	0.479	**snp38823**	Chr8	111,379,946	A > G	9.09 × 10^− 04^	0.1
snp38199	Chr21	37,251,654	G > A	4.00 × 10^− 04^	0.486	**snp21699**	Chr20	26,754,673	G > A	9.24 × 10^− 04^	0.471
snp21898	Chr17	3,285,568	A > G	4.11 × 10^− 04^	0.171	**snp24815**	Chr1	12,394,378	G > A	9.60 × 10^− 04^	0.429
snp48295	Chr8	25,933,538	C > A	4.03 × 10^− 04^	0.129	**snp45869**	Chr14	168,999	G > A	9.64 × 10^− 04^	0.386
snp14725	Chr4	15,500,675	G > A	4.28 × 10^− 04^	0.386	**snp46986**	Chr3	102,101,194	A > G	9.85 × 10^− 04^	0.379

#### Allele variation impact on tolerance to heat stress

To investigate the effect of various alleles associated with SNPs related with tolerance to HS, we compared between alleles impact on the trait. The results revealed that twenty-eight markers-SNPs exhibited a comparable effect on tolerance to HS, with maximum production of 304.90, 354.15, 291.95, and 366.95 unit, respectively, for snp12133, snp23366, and snp47446. Variations in the allele frequency were observed in multiple SNPs that influenced tolerance to HS in Egyptian goats, most of these SNPs are caused by the GA mutation. The box-plot analysis indicated significant differences between alleles in their effect on tolerance to HS, narrowing down the location of heat HS controlling loci to a few chromosomal regions. The genes where these markers are found may help the animals endure difficult circumstances, making them more tolerant to HS.

**Fig. 3:**
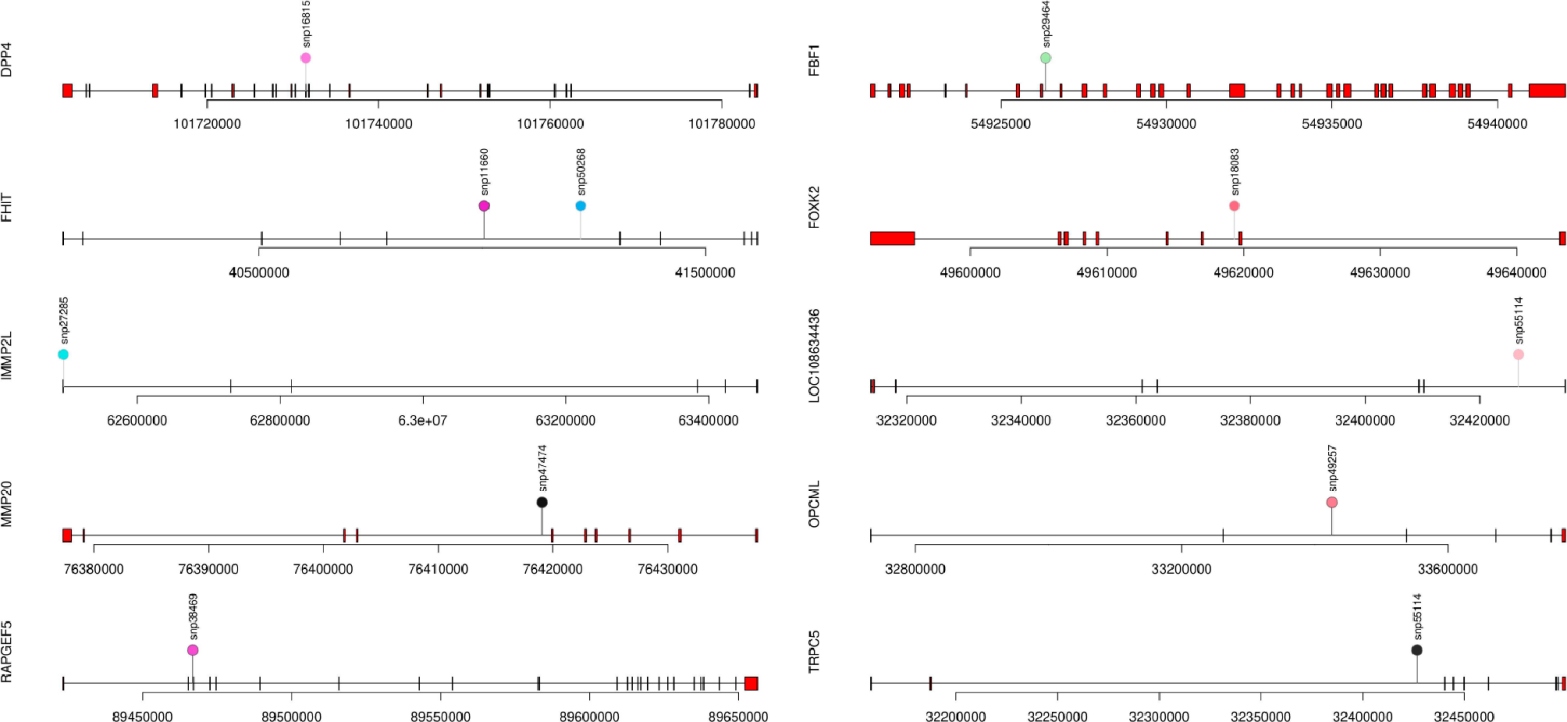
The location of SNPs associated with heat stress (HT) in goat gene structures

#### Trait-linked SNP effect on gene structure

The impact of allele variation on gene structure and function was investigated through SNP effect analysis. This analysis involved examining the interaction between SNPs alleles’ variation and gene structure, referred to as events. The impact of SNPs associated with tolerance to HS on gene structures was analyzed, and various types of interactions were observed. A small number of interactions were detected with exons (0.36%) and gene upstream regions (3.6%). Most interactions are related to introns (35.2%) and transcripts (36.36%) (Fig. 3). This analysis suggests that SNPs may have a greater influence on gene expression than on gene structure.

#### SNP associations with genes

**Fig. 4:**
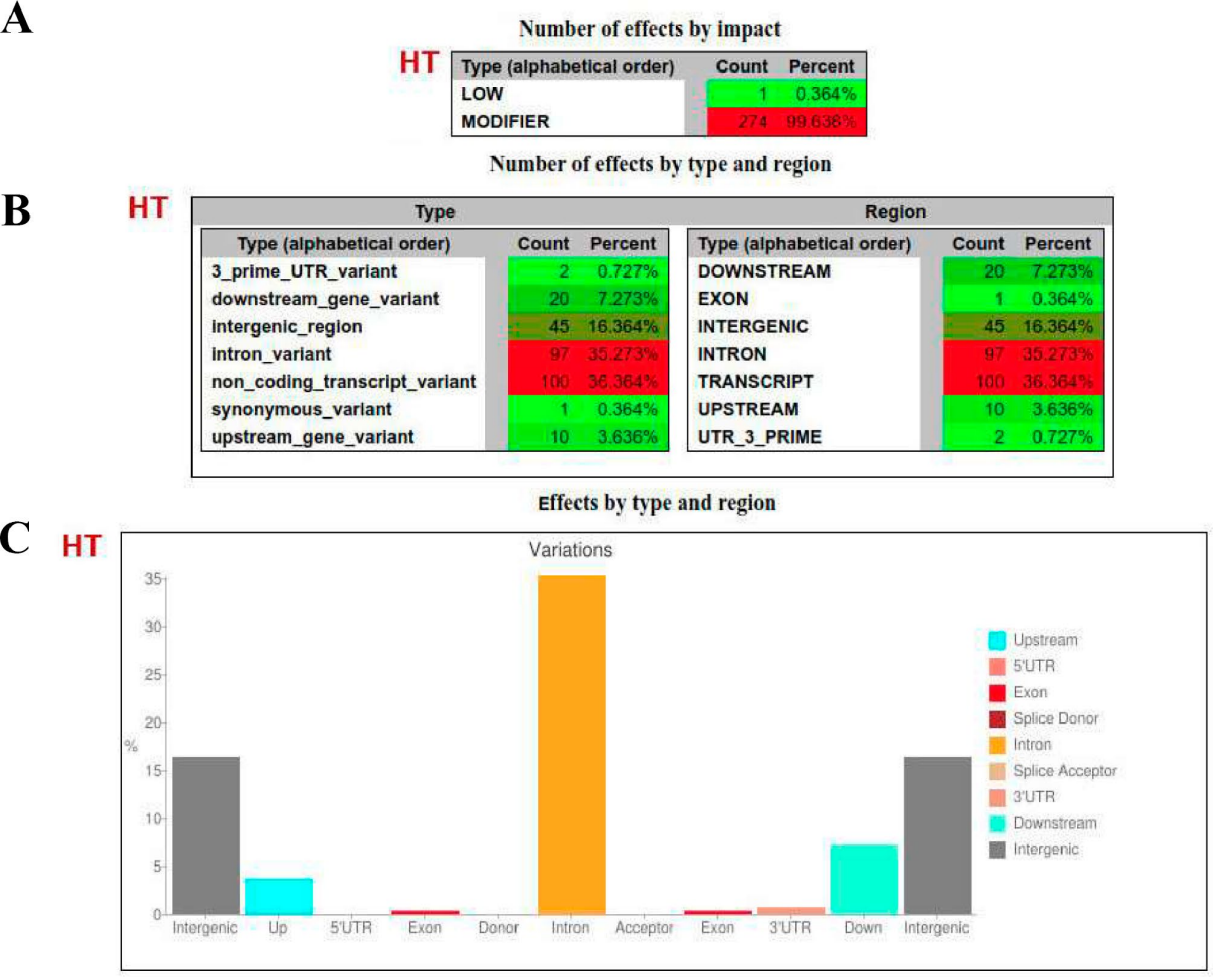
The SNP effect of trait-associated variations on the gene structure as reported by the SNPeff tool for heat stress (HS) tolerance

The SNPs associated with tolerance to HS were found in a region of several genes (Table 6; Figs. 3 and 4). The linked SNPs are associated with 45 genes, with *NAALADL2* gene having two SNPs (snp22581 and snp22586). Thirteen genes (*KDM6A*, *TRPM3*, *USP54*, *GLTSCR2*, *NAALADL2*, *GATAD2A*, *CTNNA2*, *LOC102175876*, *ZBTB8A*, *ETNPPL*, *LRRC43*, *SNTB1*, and *RPS6KA5*) have a direct SNP influence on modifying transcripts. SNP effects analysis of genes related to HS tolerance revealed that snp19073 had the most influence on the *KDM6A* gene with several impacts on the gene structure. Meanwhile, snp43427 caused seven SNP-effects on both *TRPM3* and *USP54* genes, while snp36076 caused seven events on *USP54*. Further investigation on significant SNPs and their effects on genes indicating that snp16282, snp28114, and snp22581, were among the most related SNPs to HS tolerance (with log *p*-values of 4.62, 4.47, and 3.76), affected GLTSCR2, *GALNT18*, and *NAALADL2* genes.

**Table 6:** Genes and their SNPs associated with tolerance to heat stress (HS)

SNP	Gene	SNP	Trait
snp48295	ADAMTSL1	snp22581	NAALADL2
snp59658	ARHGAP26	snp22586	NAALADL2
snp48343	CTNNA2	snp48788	RANBP17
snp27449	CTTNBP2	snp36644	RAPSN
snp20241	CXCL16	snp18981	RPS6KA5
snp55420	EFHB	snp11765	SDK1
snp23098	EMC10	snp23746	SFXN5
snp49160	ETNPPL	snp12564	SLC22A8
snp23098	FAM71E1	snp40146	SLC2A3
snp55332	FBXL4	snp12364	SMAD1
snp28114	GALNT18	snp45869	SNTB1
snp9053	GATAD2A	snp38424	SYMPK
snp7980	GATSL2	snp15472	TENM4
snp15096	GLTSCR2	snp45768	TPPP
snp25209	GPR39	snp38199	TRNAS
snp56754	GRM8	snp20506	TRNAW
snp51992	GUCY1A2	snp43427	TRPM3
snp53002	ICE2	snp21898	TTC28
snp16805	KCNH7	snp36076	USP54
snp19073	KDM6A	snp27851	XPNPEP1
snp14725	KIAA1147	snp14115	ZBTB8A
snp2427	LRRC43	snp20241	ZMYND15
snp23098	MYBPC2	snp38828	ZNF706

### Principle component analysis (PCA)

**Fig. 5:**
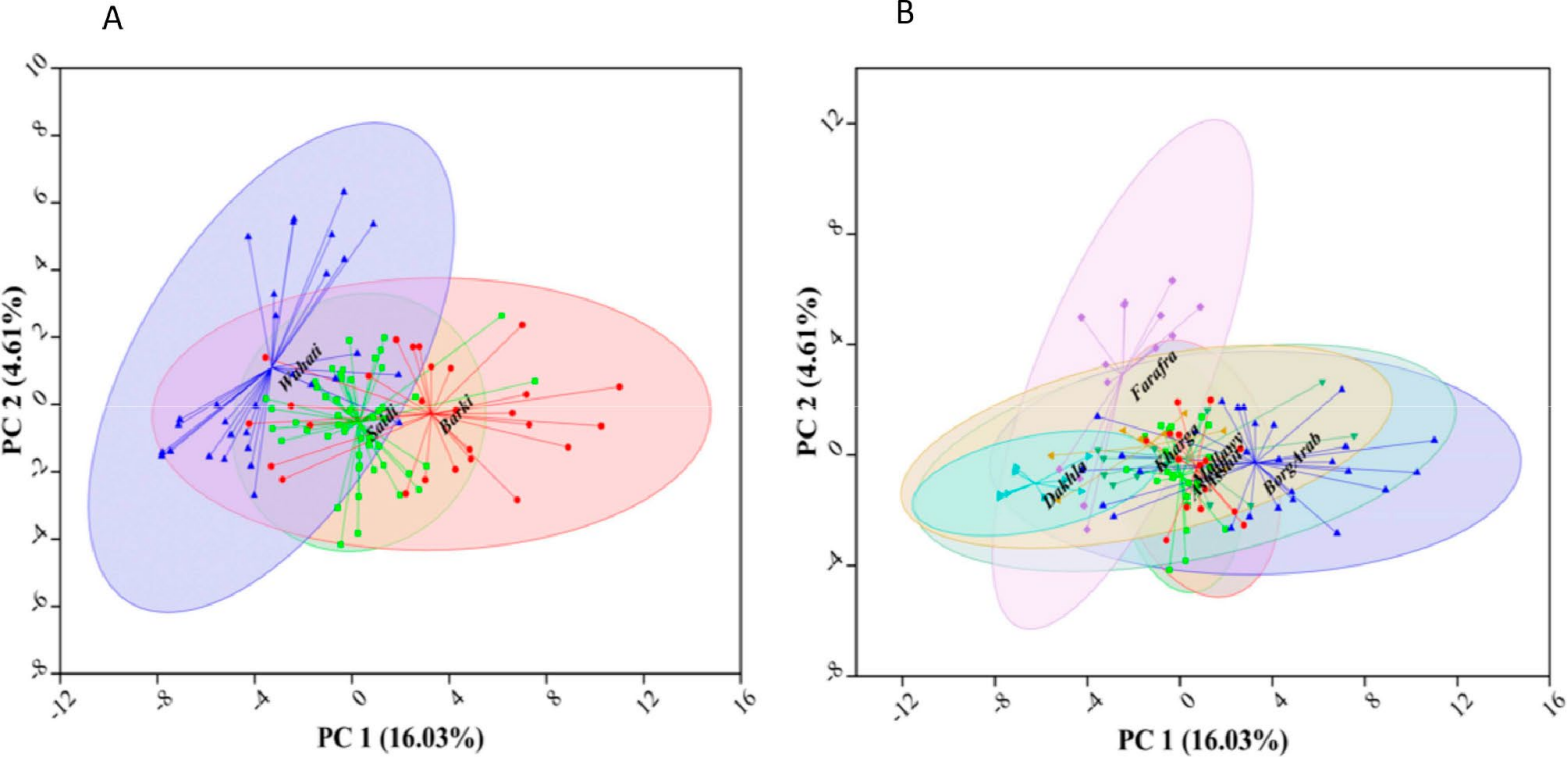
Principal Component Analysis of 108 Egyptian goats, based on the significant 90 SNPs associated with animal heat tolerance index (AHTI); (**A**) PCA for breeds and (**B**) PCA for locations

Genetic variations within breeds and locations were assessed based on the significant 90 SNPs associated with animal heat tolerance index (AHTI) using principle component analysis (PCA). Figure 5 shows that PC1 represents 16.03% and PC2 represents only 4.61% of the detected variance, which showed a low ability to discriminate genetic variation in tolerance to heat stress among the studied populations based on breed and location. At the breed level, Saidi goat is located between Wahati and Barki breeds, and Saidi goats are closer to Barki goat than Wahati regarding their tolerance to HS. Based on the locations, three groups were detected; the first group includes Kharga, Farafra, and Dakhla (New Valley, Wahati), the second group includes Assuit, Mallawy, and Aswan (Upper Egypt, Saidi) and the third group includes Borg Arab (Costal Zone Western Desert (CZWD), Barki). Saidi goats from Upper Egypt is closer to each other than Wahati goats from New Valley and Upper Egypt goats is closer to CZWD goats than New Valley goats.

### SNP networks analysis of heat stress

**Fig. 6:**
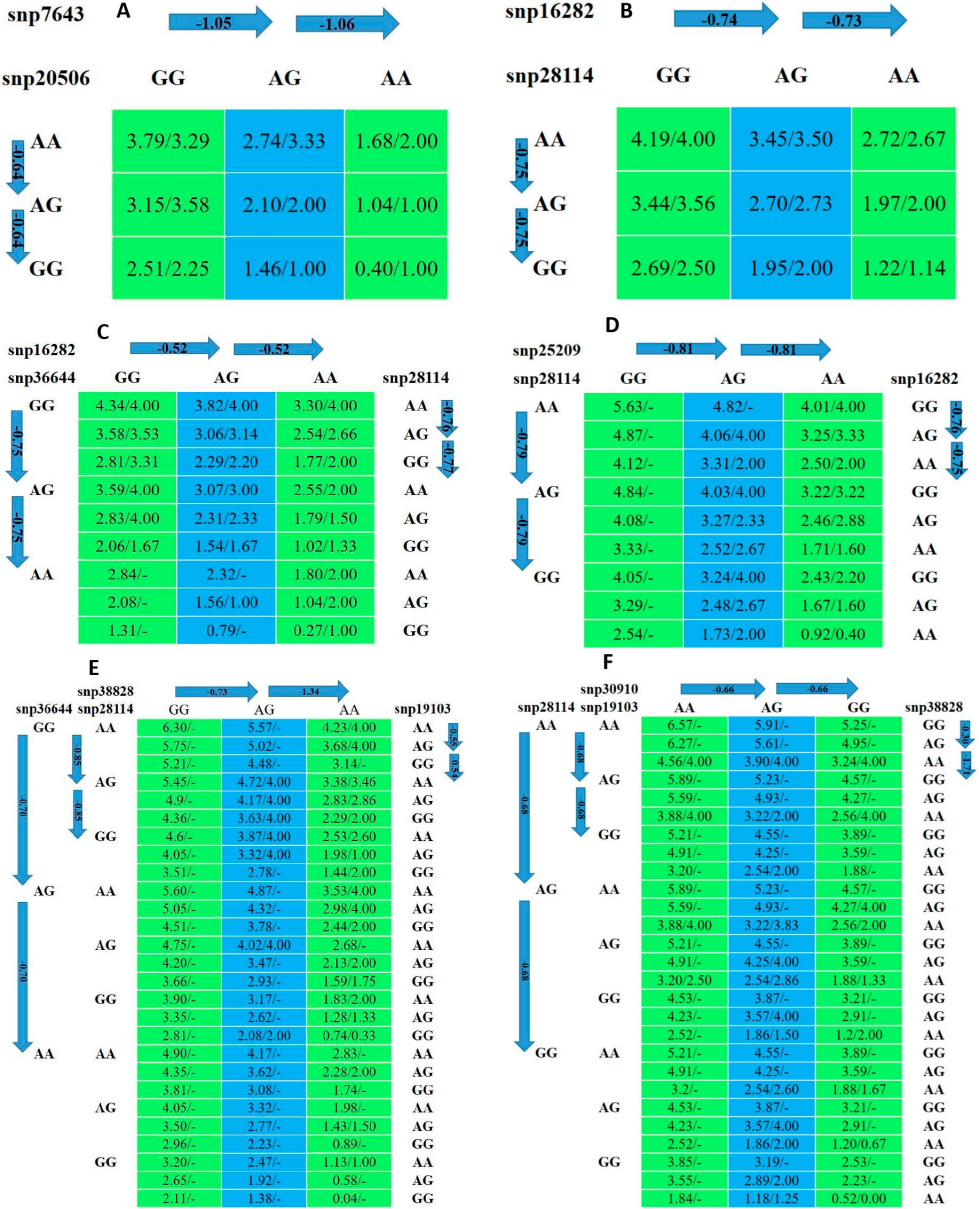
Genetic networks with multiple SNPs for animal heat tolerance index (AHTI). (**A**) SNP-network 1, (**B**) SNP-network 2, (**C**) SNP-network 3, (**D**) SNP network 4. (**E**) SNP network 5. (**F**) SNP network 6

According to [30], linear regression model with pairwise comparison method was used to identify SNP-SNP combinations. All significant SNPs were involved in multiple regression model analysis, which verified single-SNP associations and revealed more than 120 types of SNP networks for tolerance to heat stress. The simplest three types of genetic networks with multiple SNPs for animal heat tolerance index (AHTI) are shown in Fig. 6. Two SNP-networks for SNP7643 and SNP20506 (SNP-network 1), two SNP-networks for SNP28114 and SNP16282 (SNP-network 2), three SNP-networks for SNP28114, SNP36644 and SNP16282 (SNP-network 3), three SNP-networks for SNP28114, SNP16282 and SNP25209 (SNP network 4). Four SNP-networks for SNP38828, SNP19103, SNP28114 and SNP36644 (SNP network 5) and four SNP-networks for SNP38828, SNP19103, SNP28114 and SNP30910 (SNP network 6). The major changes in AHTI were approximately two values on average and are due to the substitution of GG–AA homozygotes at SNP38828 locus (SNP network 5 and 6) and at SNP7643 locus (SNP network 1). Substitution of AA-GG homozygotes changed AHTI by 1.36 (SNP network 6) to 1.7 (SNP network 5) at SNP28114 locus. On the contrary, substitution of GG-AA at SNP16282 locus changed AHTI by around 1 (SNP network 3) to 1.5 (SNP network4). Substitution of AA–GG homozygotes at SNP19103 locus changed AHTI by 1.1 (SNP network 5) to 1.36 (SNP network 6). AHTI changed by 1.4 (SNP network 5) to 1.5 (SNP network 3) owing to the substitution of GG–AA at SNP36644 locus.

Gene enrichment analysis has revealed the functional relevance of the identified genes across various pathways in animals and other biological systems. Certain genes are strongly associated with arterial stiffness, while others exhibit upregulation in specific neuronal populations, such as interneurons. Notably, some genes are linked to albumin levels in urine, indicating potential roles in renal function and systemic health. Pathways related to gene expression dynamics, such as upregulation or downregulation neuronal or cellular types (e.g., Foxp2/Olfm3 neurons and Pvalb/Th interneurons), provide further insight into cell-specific gene regulation under different conditions. Importantly, several genes are implicated in heat stress response, including NFKB1, which plays a pivotal role in inflammation and oxidative stress pathways triggered by heat exposure. Heat stress is also known to affect myelin production and maintenance, thereby impairing cellular processes crucial for broader stress adaptation. For example, myelination processes are sensitive to environmental stressors, and disruptions in myelin integrity can influence the nervous system’s ability to maintain function and repair itself under adverse conditions [48]. These findings highlight the interconnectedness of stress responses, inflammation, and cellular homeostasis, demonstrating the multifaceted impact of heat stress on both plant and animal systems.

## Discussion

High diversity is considered a promising way to modify animal production systems to mitigate concerns events, facilitate the agro-ecological transition and help livestock to adapt the potential risks [31, 32]. In the present study, the effects of hot dry climatic conditions on local Egyptian goats, namely Saidi, Barki, and Wahati breeds, collected from 7 harsh hot dry regions were examined. We focused on identifying genomic loci that could potentially be utilized to monitor and control breeds’ tolerance to HS, while maintaining their production. It is one of the few studies that utilized SNP marker technology to identify genes linked with tolerance to HS in goats. The SNP genotyping has several advantages over the marker systems, including the ability to process large populations [33]. It also enables powerful approaches such as genome-wide association studies, and the identification of genes responsible for complex disorders [33, 34].

Genomic analysis of heat stress tolerance identified 90 marker SNPs modifying the structure of 45 different genes across the genome (Table 5; Figs. 2, and 3). Gene annotation analysis was used to identify the related genetic variant affecting tolerance to HS. The presence of numerous genes linked with tolerance to HS implies that they are likely polygenic, with multiple genomic loci impact. This polygenic feature is particularly noticeable, with several genes demonstrating significant association with the breeds’ response to rising temperatures in their environment. Genes linked to climate resilience have been identified through the examination of the genomic regions. The genomic architecture of the animal’s response to HS is complex due to the extensive impact of HS on their biological systems. This response likely involves the activation of numerous biological pathways to help animals to cope with the adverse effects of HS.

The data analysis revealed several genes that are associated with tolerance to HS. It is worth noting that most of these genes have multiple functions and may be involved in more than one group. We were able to categorize the potential genes into three main groups, include those involve in response to HS (*USP54*, *KDM6A* and *ETNPPL*), stress response (*USP54*, *ETNPPL*, *GLTSCR2*, and *NAALADL2*), feed intake (*TRPM3* and *ZBTB8A*), and reproduction (*KDM6A*, *GATAD2A* and *ETNPPL*). This categorization provides useful ways to understand the potential role of these genes in the tolerance to HS in subtropical goats.

The first group are the genes involved in response to and are often associated with key biological processes such as reproduction, immune response, and metabolism, which is given their interconnected nature. For example, the *USP54* gene was found to be located in genomic regions that control HS response in cattle [35]. This gene also plays a significant role in various biological processes such as steroid biosynthesis and metabolism, cell-cell signaling, ion transport and homeostasis, cell growth, and stress response [36, 37] Another gene, *KDM6A* was reported to contribute to variation in immune phenotypes and HS response [38]. It encodes a histone demethylase that is associated with litter size and fertility traits in goats [39, 40]. The *ETNPPL* gene was identified as a potential for HS interactions with milk production in dairy cattle as it activates various biological processes in animals. Finally, *GATAD2A* gene acts as a neurobiological brake that modulates the GnRH pattern pulse which is necessary for normal reproduction. It also regulates the repressive action of Mbd3/NuRD during the reprogramming process [41, 42]. These findings provide insights into the potential roles of these genes in tolerance to HS in subtropical goats.

The second group of genes is associated with the immune response, includes *GLTSCR2* and *NAALADL2*. *GLTSCR2* play a role in the immune response against viral infections [43]. On the other hand, *NAALADL2* is involved in maintaining immune homeostasis in animals through number of variations, as demonstrated in African goats [44, 45]. Finally, the other group of genes are potentially controlling animal feed intake, such as. *TRPM3* and is associated with animal appetite (feed efficiency) [46]. *ZBTB8A* is involved in binding DNA and transcriptional regulation and is potentially involved in residual feed intake, dry matter intake, feed efficiency, feed conversion, body weight gain, and residual intake in Nellore cattle [47].

The interplay between these SNPs and physiological features suggests that genetic variants can have a direct impact on an animal’s ability to cope with heat stress. Goats with favorable alleles in genes such as *KDM6A* and *TRPM3* may produce more heat shock proteins and regulate their temperature more efficiently, resulting in better performance and survival in hot environments. *KDM6A* is gene encodes a histone demethylase, which regulates gene expression by modifying chromatin structure, *KDM6A* gene regulate heat shock proteins expression and other stress-responsive genes, which are essential for maintaining cellular homeostasis during heat stress. By regulating the expression of these protective proteins, *KDM6A* can promote goats’ physiological tolerance to high temperatures [48]. Similarly, *TRPM3*, gene involved in ion channel function, may influence cellular homeostasis and thermoregulation, thereby reducing the physiological effects of heat stress [49]. The discovery of these genetic markers provides valuable insights into the biological mechanisms of heat tolerance. Integrating physiological and genomic data allows better understanding the complex relationships that contribute to breed-specific variability in heat stress resilience. This knowledge can be applied to develop breeding programs aiming at enhancing heat tolerance in subtropical goat breeds, and improving their adaptability to changing climatic conditions.

The integration of gene expression studies and pathway analysis in these contexts underscores the importance of stress-responsive genes not only in protecting cells from damage but also in regulating key physiological processes, such as immune response, energy metabolism, and tissue repair. These genes, particularly involved in heat stress responses like *NFKB1*, which can serve as critical biomarkers for understanding how organisms adapt to environmental challenges. The identification of such genes opens the possibility of employing them in marker-assisted selection (MAS), a powerful tool in both agricultural and medical research. By selecting for these markers, it is possible to develop plant varieties with enhanced resilience to abiotic stresses, such as heat or drought, and improve livestock with better stress tolerance and health outcomes. Similarly, in medical applications, stress-responsive genes could guide therapeutic interventions to mitigate damage in stress-related conditions. These insights not only advance our understanding of cellular resilience but also provide practical avenues for applying genetic knowledge to enhance sustainability and adaptability in various biological systems.

The studied subtropical regions (Upper Egypt, Coastal Zone of Western Desert, and New Valley Desert Oasis) has different hot climatic conditions, including temperature, humidity, and solar radiation intensity. These environmental differences may have contributed to the variation in the expression of tolerance to HS observed among the studied goat populations [50]. Regions with scant or low-quality vegetation may enhance the physiological stress faced by the animals, potentially influencing their genetic response to HS [51]. Furthermore, management practices, such as grazing patterns, shelter availability, and water provision, can influence the animals’ ability to cope with HS. Differences in these practices between the studied regions may have resulted in variations in the physiological parameters measured and the genetic markers identified [2]. Considering these factors into account is critical for interpreting the study’s findings and emphasized the importance of specialized management strategies for improving HS tolerance in subtropical goat populations. Future research should investigate the interactions between environmental conditions, management practices, and genetic factors in order to develop comprehensive approaches to enhance HS resilience.

While the study sheds light on the genetic foundations of tolerance to HS in subtropical Egyptian goats, some limitations can be acknowledged. While sample sizes reflect the demographic distributions of the breeds in the country, the disparity may skew the results. Barki goats with larger sample size may provide more statistical power than Saidi goats with smaller sample size. Future studies should aim for more evenly distributed sample sizes, and include goat populations from different geographical regions to validate the results across multiple breed populations, and confirm the roles of the identified genes and SNPs in tolerance to HT by functional studies.

## Conclusion

The present data identified several genes that are associated with tolerance to HS in three population of subtropical Egyptian goats. Most of these genes have multiple functions and are involved in more than one performance criteria, e.g. reproduction, immune response, and metabolism, logically given their interconnected nature. Identifying these genomic loci can potentially benefited breeding programs aims to promote tolerance of subtropical goat to HS, besides improving their production performance. Marker-assisted selection (MAS) can be employed to identify individuals with favorable alleles for improving the overall resilience of the population to HS. Studies to enhance our understanding of the genetic architecture underlying differences in response between subtropical and temperate goats breeds and their crossbreeds are needed. Selective breeding for specific traits, such as heat tolerance, may unintentionally limit genetic diversity within the populations, decline in diversity may render animals more vulnerable to diseases and other environmental changes. It’s critical to strike a balance between selection for heat stress tolerance and preservation of genetic diversity to ensure long-term health and resilience of livestock populations.

## Electronic supplementary material

Below is the link to the electronic supplementary material.

Supplementary Material 1

Supplementary Material 2

## Data Availability

The datasets generated and analyzed from the study can be found in the following online repository (https://zenodo.org/records/14885406).
