# Supplementary material for: Genomic analysis association of tolerance to heat stress in subtropical Egyptian goats Raised in hot dry environment
Source: BMC Genomics. 2025 Jun 11;26:568. doi: 10.1186/s12864-025-11748-x (PMC12153181; doi:10.1186/s12864-025-11748-x)
Supplement: Supplementary file 1 — Supplementary Material 1 [file 12864_2025_11748_MOESM1_ESM.docx]

**Table S1: Longitudinal climatic data of three different regions under study.**

|  |  |  | Western Desert | New Valley | Upper Egypt |
| --- | --- | --- | --- | --- | --- |
| Climate zone |  |  | subtropics | dry desert climate | subtropics to tropics |
| Latitudes |  |  | 31° 21' N to  25° 27' N | [24°32′44″N 27°10′24″E](https://geohack.toolforge.org/geohack.php?pagename=New_Valley_Governorate&params=24_32_44_N_27_10_24_E_region:EG-WAD_type:adm1st) | 29° 29' N to  22° 21' N |
| Average temperatures | July | Day | 36.3 | 38.5 | 40.5 |
|  |  | Night | 23.6 | 26.04 | 25.8 |
|  | August | Day | 36.4 | 39 | 40.7 |
|  |  | Night | 24.1 | 26.7°C | 26 |
| Average humidity | July |  | 48 | 21 | 31 |
|  | August |  | 50 | 23 | 34 |
| Average THI | July |  | 42 |  | 43 |
|  | August |  | 42 |  | 44 |

THI: [Temperature–humidity index. Source: https://www.worlddata.info/climate and https://weatherandclimate.com.](C:\\Users\\Dr.Adel\\Downloads\\Temperature–humidity index. Source: https:\\www.worlddata.info\\climate and https:\\weatherandclimate.com)
